# Research on the business performance evaluation method for small and medium-sized enterprises in cross-border e-commerce based on artificial bee colony optimized LSTM model

**DOI:** 10.1038/s41598-025-17435-x

**Published:** 2025-08-28

**Authors:** Rundong Liao, Yuxi Chai

**Affiliations:** 1 School of Digital Commerce, Zhejiang Polytechnic University of Mechanical and Electrical Engineering, Hangzhou, 310053 Zhejiang China; 2 Ningbo University DongHai Academy, Ningbo, 315211 Zhejiang China

**Keywords:** Artificial bee colony algorithm, Long short-term memory network, Cross-border e-commerce SMEs, Business performance evaluation, Engineering, Mathematics and computing

## Abstract

The rapid development of the cross-border e-commerce industry has posed challenges for small and medium-sized enterprises (SMEs), such as large fluctuations in the international market environment and limited resource allocation, which increases the uncertainty and complexity of their business performance. Traditional business performance evaluation methods are inadequate in handling complex nonlinear data and real-time responsiveness, making them difficult to meet the demands of a dynamic market environment. Based on data from 10 cross-border e-commerce SMEs from 2020 to October 2023, this paper proposes a business performance evaluation method based on the Artificial Bee Colony Optimized Long Short-Term Memory Neural Network (ABC-LSTM) to improve the predictive accuracy of complex time series data analysis and the model’s generalization ability. The ABC-LSTM model outperforms GA-LSTM, XGBoost, and traditional LSTM models in performance metrics such as Mean Squared Error (0.037), Mean Absolute Error (0.016), and Time Dependency Error (0.019), demonstrating faster convergence speed and higher stability. Additionally, this study analyzes the hierarchical characteristics of performance among different enterprises, revealing the advantages of high-performance enterprises in resource integration, supply chain management, and market expansion, as well as the bottleneck issues of low-performance enterprises. The results validate the significant advantages of the ABC-LSTM model in evaluating the business performance of SMEs in cross-border e-commerce. It not only improves the accuracy of multi-dimensional business data analysis for cross-border e-commerce enterprises but also provides a scientific basis for enterprises in resource integration, supply chain management, and market expansion.

## Introduction

As an important form of global trade, cross-border e-commerce has become a key path for small and medium-sized enterprises (SMEs) to expand into international markets. However, these enterprises commonly face challenges such as the high complexity and volatility of business performance evaluation, with traditional methods struggling to accurately capture their dynamic characteristics, thereby limiting the effectiveness of resource optimization and strategic formulation^1–3^. Although deep learning techniques have demonstrated potential in time series analysis, existing models still have shortcomings in hyperparameter optimization and global adaptability, making them inadequate to meet the practical needs of SMEs in cross-border e-commerce^4,5^. Therefore, this study proposes an LSTM model combined with an Artificial Bee Colony Optimization (ABC) algorithm, aiming to improve the accuracy and stability of performance evaluation, thus supporting enterprises in optimizing resource allocation and formulating scientific strategies.

Existing research has shown a diverse range of exploratory paths in the field of business performance evaluation. Diao & Zhang^6^ explored the application of data mining techniques in performance management for SMEs, pointing out that decision tree-based and clustering algorithms can effectively extract key patterns from business data. Sako et al.^7^ analyzed the performance of deep learning models in macroeconomic data forecasting, discovering that Recurrent Neural Networks (RNNs) have potential in processing long time series data. Sadik et al.^8^ attempted to combine Bayesian networks with traditional statistical models, proposing an integrated performance prediction method. However, in high-dynamic data scenarios, the model’s generalization ability still remains insufficient. While these studies provide important technical support for business performance evaluation methods, existing research still faces issues in adapting to multi-dimensional dynamic data, low model optimization efficiency, and incomplete evaluation dimensions in the context of cross-border e-commerce SMEs, which exhibit high dynamics and data complexity. To address these shortcomings, this research proposes a hybrid model, ABC-LSTM. The Artificial Bee Colony (ABC) algorithm, by simulating the foraging behavior of bees, achieves a dynamic balance between global search and local optimization, significantly improving the efficiency and adaptability of hyperparameter optimization. Combined with LSTM’s ability to model long-term dependencies in time series data, the ABC-LSTM model can more effectively capture the characteristics of multi-dimensional dynamic business data for SMEs in cross-border e-commerce. Through empirical analysis of data from 10 cross-border e-commerce SMEs from 2020 to 2023, this study validates the model’s superiority in complex dynamic scenarios. The research results indicate that the ABC-LSTM model significantly outperforms traditional models in terms of prediction accuracy, stability, and adaptability, providing enterprises with a more comprehensive performance evaluation tool and decision support. This research not only addresses the limitations of existing studies in model optimization and adaptability under high-dynamic data scenarios but also provides practical evidence for the accurate evaluation of key dimensions such as profitability and asset operation efficiency in cross-border e-commerce SMEs. The proposed model offers scientific support for enterprises in critical areas such as resource integration, supply chain management, and market expansion, thereby enhancing SMEs’ capacity to cope with real-world challenges such as international market volatility and order uncertainty.

The academic contributions of this study are reflected in the following three aspects: First, it constructs a hybrid model combining the Artificial Bee Colony optimization mechanism with the LSTM architecture, enhancing the hyperparameter tuning capability and stability of deep learning models in high-dynamic environments, and expanding the application path of intelligent optimization algorithms in enterprise management modeling. Second, by introducing multidimensional metrics such as Time Dependency Error into the model evaluation framework, the study achieves fine-grained characterization of performance fluctuation trends, improving the adaptability and interpretability of complex time-series modeling. Third, by focusing on cross-border e-commerce SMEs as the research subject, the study responds to the insufficient attention in existing literature to performance evaluation in high-volatility industries, enriching the theoretical foundation of this field in terms of modeling approaches and application scenarios.

The structure of this paper is arranged as follows: The first section introduces the research background, problems, and significance; the second section provides a literature review, summarizing existing research on intelligent algorithms in business performance evaluation; the third section describes the research design, including data sources, indicator system, model design, and evaluation metrics; the fourth section presents empirical analysis and results to validate the superiority of the proposed model; the fifth section is a discussion, summarizing the research findings and comparing them with other scholars’ work. The final section concludes the study, summarizing the results and suggesting directions for future research.

## Literature review

### Application of deep learning models in business performance evaluation

Deep learning algorithms have gained widespread application in recent years for business performance evaluation due to their superior feature extraction and nonlinear modeling capabilities. Compared with traditional regression methods, they are more adept at handling high-dimensional, multivariable, and structurally complex data, especially in highly dynamic, time-dependent business environments, where they demonstrate stronger adaptability^9^. In business contexts, deep learning not only identifies long-term dependencies hidden within data but also captures the interactive effects among different operational variables, gradually becoming an essential tool in performance analysis.

Among the various deep learning architectures, Long Short-Term Memory (LSTM) networks have been widely employed for forecasting financial data and business performance due to their advantages in time-series modeling. Nong^10^ developed a multi-layer LSTM-based financial risk prediction model, and experimental results showed that this structure significantly improved prediction accuracy, although overly deep networks led to a decline in training efficiency. To strike a balance between accuracy and efficiency, Cho & Lee^11^ confirmed the effectiveness of the structure in handling financial time-series data, particularly in volatile and information-asymmetric scenarios. Recognizing the varying importance of different variables to business outcomes, Zhu et al.^12^ used a Gated Recurrent Unit (GRU) structure to predict monthly sales data of retail enterprises. Their results showed that GRU maintained strong time-series modeling ability while effectively reducing model complexity and computational cost. In more complex time-series tasks, Bi-LSTM, as an extension of LSTM, introduces bidirectional propagation to capture contextual information and enhance prediction completeness and robustness. The Bi-LSTM performance prediction model proposed by Jiang et al.^13^ demonstrated excellent results in modeling SME operational data. Wen & Li^14^ integrated attention mechanisms into LSTM to guide the model in focusing on key input features, significantly improving prediction accuracy and generalizability for multivariable time-series datasets.

In recent years, some studies have also explored incorporating external macro-environmental variables into model inputs to enhance robustness. Chen et al.^15^ introduced external indicators such as exchange rates and inflation rates into SME performance modeling and developed a multi-channel input structure based on LSTM, effectively mitigating the performance degradation caused by external shocks. Reference^16^ combined Empirical Mode Decomposition (EMD) with LSTM to improve the model’s ability to handle non-stationary sequences. Similarly, Jahin et al.^17^ employed a CNN-LSTM architecture in supply chain performance forecasting, using Bayesian optimization to fine-tune model parameters, and achieved superior results compared to traditional deep learning models across multiple evaluation metrics.

Despite substantial progress in applying deep learning to business performance evaluation, challenges remain in real-world deployment. On one hand, deep learning models are highly sensitive to parameter initialization and hyperparameter settings, making them prone to local optima. On the other hand, their considerable computational demands limit widespread adoption among SMEs. Therefore, improving training efficiency and stability, while enhancing adaptability to dynamic economic conditions, is an important research direction and provides the methodological basis for introducing intelligent optimization algorithms and fusion strategies.

### Application of intelligent optimization algorithms in business performance evaluation

Intelligent optimization algorithms, known for their strong global search capabilities and adaptive parameter tuning, have become key tools for improving the performance of business evaluation models. Compared with traditional manual tuning methods, these algorithms can effectively explore high-dimensional parameter spaces, avoid local optima, and enhance both prediction accuracy and model robustness. In recent years, researchers have widely applied intelligent optimization strategies such as Genetic Algorithms (GA), Particle Swarm Optimization (PSO), and Artificial Bee Colony (ABC) algorithms to business performance modeling, showing remarkable advantages especially in optimizing parameters for deep learning models.

Zahedi et al.^18^ combined the ABC algorithm with Support Vector Regression (SVR) to construct a financial health evaluation model for enterprises, which significantly improved both predictive accuracy and robustness. Chen & Du^19^ applied PSO to neural network modeling of enterprise supply chain networks, effectively accelerating convergence and enhancing model performance. Building on this, Oladipo et al.^20^ introduced a dynamic weight adjustment mechanism to PSO, which further enhanced the convergence stability of the algorithm on time-series datasets. Other studies have explored combining optimization algorithms with ensemble modeling strategies to improve the practical value of models in real-world applications. For instance, Wang et al.^21^ constructed an ensemble prediction framework integrating LSTM and Random Forest (RF) for sales data analysis and replenishment strategy optimization, significantly improving usability and response efficiency. Li et al.^22^ proposed integrating K-means clustering with the Artificial Bee Colony algorithm to reduce parameter redundancy in high-dimensional data scenarios, thereby improving overall optimization efficiency.

From a methodological perspective, intelligent optimization algorithms dynamically balance global exploration with local fine-tuning, preventing deep models from experiencing early convergence or performance bottlenecks due to suboptimal configurations. Esmaeili et al.^23^ proposed an agent-based collaborative random search mechanism, which demonstrated enhanced exploration capacity and precision in the hyperparameter space, further expanding the applicability of optimization algorithms. In business performance modeling tasks, such strategies help enhance model stability and robustness, especially in SME environments characterized by frequent variable fluctuations and cross-period changes. Moreover, the integration of optimization strategies with deep architectures continues to push practical boundaries. Onorato^24^ applied Bayesian optimization to fine-tune neural networks (especially CNNs), using Gaussian Process regression and EI/UCB acquisition functions to reduce tuning iterations and improve model accuracy.

Overall, intelligent optimization algorithms provide strong support for enhancing deep learning models in business performance prediction. Their global search capabilities and tuning efficiency offer clear practical value, particularly under conditions of small sample sizes and complex indicator systems, as often seen in SMEs. Nonetheless, existing optimization methods still face challenges such as high computational costs and sensitivity to initial configurations. Future improvements are needed in areas such as convergence efficiency, intelligent initialization strategies, and the integration of optimization with interpretability mechanisms.

### Research progress of hybrid models in business performance evaluation

To leverage the modeling strengths of deep learning and the tuning advantages of intelligent optimization algorithms, researchers have increasingly explored hybrid modeling approaches in recent years. These models are designed to achieve high-performance evaluations in complex business environments. Typically, hybrid models utilize optimization algorithms to dynamically tune hyperparameters in deep networks, improving convergence efficiency, enhancing stability, and reducing dependency on initial configurations—making this an important trend in performance prediction research^25^.

Sukestiyarno et al.^26^ developed a hybrid GA-LSTM model for forecasting enterprise market volatility. Their study found that genetic algorithms performed well in optimizing hidden layer size and learning rates, effectively improving long-term forecasting accuracy. Pai et al.^27^ combined a Group Search Optimizer (GSO) with LSTM for multi-step rolling forecasts of financial data, excelling in handling variable lags and nonlinear fluctuations. Ali et al.^28^ applied Empirical Mode Decomposition (EMD) for multi-scale processing of operational sequences and integrated it with LSTM to enhance model fitting for non-stationary time-series data. In terms of structural optimization, Liu & Vakharia^29^ proposed a BO-CNN-LSTM model that used Bayesian optimization to jointly tune network structure and hyperparameters, exhibiting improved generalization and training efficiency in supply chain performance modeling. Cannelli et al.^30^ further explored agent-based collaborative hybrid optimization algorithms capable of stable global searches in high-dimensional hyperparameter spaces, offering flexible support for designing complex network structures.

Despite the progress in improving predictive accuracy and model stability, most existing hybrid model studies focus on traditional industries such as manufacturing and finance, with limited research on SMEs in the cross-border e-commerce sector. Due to their international business models and highly dynamic market environments, cross-border e-commerce SMEs face challenges such as large order fluctuations, complex supply chains, and constrained resource allocation. These characteristics impose higher demands on real-time responsiveness, adaptability, and multi-objective analysis in performance evaluation models. To address these issues, this paper focuses on cross-border e-commerce SMEs and proposes a business performance evaluation method based on an Artificial Bee Colony Optimized LSTM model. By leveraging the global search and local exploration strategies of the ABC algorithm, the model efficiently optimizes LSTM hyperparameters, enhancing predictive accuracy and adaptability under limited resources and volatile market conditions. Moreover, the introduction of multi-dimensional performance indicators allows for the construction of an evaluation framework more aligned with the characteristics of SMEs.

## Research design

### Data sources and preprocessing

The data used in this study is sourced from publicly available information channels such as the Guotai’an database, Giant Tide Information Network, and company annual reports, covering quarterly financial and operational performance data of 10 small and medium-sized enterprises (SMEs) engaged in cross-border e-commerce from the first quarter of 2020 to the fourth quarter of 2023. The selection of sample enterprises was based on several criteria, including a high proportion of cross-border e-commerce as their core business, availability of complete financial data, diversity in operational models, and representativeness in industry structure, thereby ensuring the feasibility and adaptability of the study. As shown in Table 1, the 10 sample enterprises are located in economically active eastern coastal regions of China, including Guangdong, Zhejiang, Fujian, Jiangsu, Shanghai, and Shandong—areas with robust cross-border e-commerce development. These enterprises span multiple consumer categories such as home goods, apparel and accessories, small electronics, beauty and skincare, and baby products, and operate through various business models, including independent websites, B2C platforms, and proprietary platforms, reflecting a high degree of industry representativeness and structural diversity.

**Table 1:** Basic information of cross-border e-commerce enterprises.

Company ID	Industry	Main products	Operation mode	Location
Company A	Home goods	Furniture, storage items	Independent website + Overseas warehouse	Guangdong
Company B	Apparel & accessories	Clothing, accessories	B2C: Amazon platform	Zhejiang
Company C	Small electronics	Mobile accessories	Proprietary platform	Fujian
Company D	Home goods	Household storage	B2C: AliExpress platform	Jiangsu
Company E	Home appliances	Small appliances	Independent website + Overseas warehouse	Zhejiang
Company F	Apparel & accessories	Jewelry, footwear	B2C: Etsy + AliExpress platform	Fujian
Company G	Home goods	Stationery, kitchenware	B2C: Amazon platform	Guangdong
Company H	Beauty & personal care	Skincare products	Proprietary platform + Social commerce	Shanghai
Company I	Small electronics	Cables, batteries	Independent website	Shandong
Company J	Baby products	Baby supplies	Proprietary platform + Overseas warehouse	Guangdong

To ensure the completeness and consistency of the time series data, missing values are filled using linear interpolation or nearest-neighbor interpolation methods, while outliers are detected and removed using box plots. Additionally, all indicators are standardized using the z-score method to eliminate dimensional differences between different metrics. Given that the cross-border e-commerce industry may experience sales fluctuations during key promotional periods such as Black Friday, Double Eleven, and the Christmas season, this study conducted technical examinations on the quarterly distribution characteristics of key performance indicators during data processing. In addition, balance control measures were applied based on the time span of the sample. The dataset covers 16 quarters, which helps mitigate the impact of short-term outliers or periodic fluctuations, thereby enhancing the stability of the data series for dynamic modeling. For the convenience of model training and testing, the data set is randomly split into training and testing sets in a 7:3 ratio.

### Indicator system construction

In constructing the business performance evaluation system for cross-border e-commerce SMEs, this study comprehensively considered enterprises’ performance in terms of profitability, risk resilience, resource utilization efficiency, and market expansion potential. Six key indicators were selected, covering four dimensions: revenue level, financial stability, asset operation efficiency, and business expansion capacity. Revenue level is the most direct reflection of a firm’s operational outcomes. Within this dimension, Return on Assets (ROA) and Return on Invested Capital (ROIC) are widely used to measure profit efficiency based on enterprise resources^31^, making them highly suitable for evaluating the value-creation capabilities of SMEs under limited asset conditions. Financial stability forms the foundation for continuous business operations. In the context of highly uncertain cross-border markets, the Debt-to-Asset Ratio (DAR) and Quick Ratio (QR) help assess an enterprise’s solvency and the rationality of its capital structure^32^, playing a key role in identifying high-risk operational states. Asset operation efficiency reflects a company’s capacity to allocate internal resources. In the cross-border e-commerce sector—where inventory management and supply chain responsiveness are core competitive advantages—this dimension holds strong practical relevance^33^. Given that cross-border e-commerce SMEs must navigate constantly changing external market conditions, business expansion capacity is included as a critical dimension reflecting enterprise growth potential. Revenue Growth Rate (RGR), in particular, serves as an effective measure of a firm’s market sensitivity and expansion potential^34^. Therefore, these four dimensions—spanning both static financial structure and dynamic growth potential—form a relatively comprehensive performance evaluation framework that reflects the structural characteristics of SME performance. The detailed indicator system is presented in Table 2.

**Table 2:** Indicator system for business performance evaluation of SMEs in Cross-border E-commerce.

Indicator category	Name	Definition
Revenue level	Return on assets (ROA)	Net profit / Total assets (%)
	Return on investment (ROI)	After-tax operating profit / Total invested capital (%)
Financial stability	Debt-to-asset ratio (DAR)	Total liabilities / Total assets (%)
	Quick ratio (QR)	Quick assets / Current liabilities (%)
Asset operation efficiency	Total asset turnover (TAT)	Operating revenue / Average total assets
Business expansion capacity	Revenue growth rate (RGR)	(Current period revenue - previous period revenue) / Previous period revenue

### Model construction

To address the complexity and nonlinearity in the business performance evaluation of SMEs in cross-border e-commerce, this study proposes a hybrid model called ABC-LSTM, based on the Artificial Bee Colony (ABC) optimization algorithm and Long Short-Term Memory (LSTM) neural network. The model optimizes the hyperparameters of LSTM using the ABC algorithm, making it more effective in capturing performance variation features of cross-border e-commerce enterprises under different operating environments.

The LSTM model, due to its ability to handle the long-term dependency characteristic of time series data, is an ideal tool for solving nonlinear dynamic systems. In the model, the inputs include key business indicators of the enterprise, and the output is the predicted value of business performance. The core logic of LSTM can be described as follows: The forget gate controls the retention of the previous time-step state to determine the information transmission ratio, with the following formula:

1 $${f_t}=\sigma ({W_f} \cdot [{h_{t - 1}},{x_t}]+{b_f})$$

where${f_t}$represents the activation value of the forget gate, ${h_{t - 1}}$is the hidden state of the previous time step,${x_t}$is the current input, and ${W_f}$and ${b_f}$are the weight matrix and bias vector, with σ being the Sigmoid activation function. The input gate updates the information state for the current time step, and its expression includes the gate activation value and the candidate state:

2 $$\left\{ {\begin{array}{*{20}{c}} {{i_t}=\sigma ({W_i} \cdot [{h_{t - 1}},{x_t}]+{b_i})} \\ {{{\tilde {C}}_t}=\tanh ({W_C} \cdot [{h_{t - 1}},{x_t}]+{b_C})} \end{array}} \right.$$

where${i_t}$determines the information update ratio, $\tilde {C}$is the new candidate memory cell state, W and b are the weight and bias matrices, and tanh is the hyperbolic tangent function. The update of the memory cell state for the current time step is determined by both the forget and input gates:

3 $${C_t}={f_t} \cdot {C_{t - 1}}+{i_t} \cdot {\tilde {C}_t}$$

The output gate generates the hidden layer output based on the current memory cell state:

4 $$\left\{ {\begin{array}{*{20}{c}} {{o_t}=\sigma ({W_o} \cdot [{h_{t - 1}},{x_t}]+{b_o})} \\ {{h_t}={o_t} \cdot \tanh ({C_t})} \end{array}} \right.$$

where o_t_ is the output gate activation value. In practice, the performance of the LSTM model heavily depends on the choice of hyperparameters (such as learning rate, number of hidden units, etc.). To avoid inefficiencies caused by subjective selection, this study introduces the ABC algorithm to optimize the hyperparameters. The ABC algorithm simulates the foraging behavior of bees, combining global and local search strategies to improve parameter optimization efficiency. The fitness function is defined by the Mean Squared Error (MSE):

5 $${\text{MSE}}=\frac{1}{n}\sum\limits_{{i=1}}^{n} {{{({y_i} - {{\hat {y}}_i})}^2}}$$

where y_i_ is the actual value of the i-th sample, and ${\hat {y}_i}$ is the predicted value of the i-th sample. The optimization process involves randomly initializing a population of parameters, calculating the fitness values, and dynamically adjusting the parameter combinations. A population P of parameters is randomly generated, and a solution is assigned to each bee. For each parameter set θ∈P, the fitness F(θ) is calculated using the training data. During the foraging stage, each worker bee searches around the current solution θ\thetaθ in its neighborhood, and the solution update formula is:

6 $$\theta ^{\prime}=\theta +\phi \cdot (\theta - {\theta _{random}})$$

where ϕ is a random disturbance factor, and θ_random_ is a randomly selected solution. After several iterations, the parameter combination that maximizes fitness is output. With the optimized parameters, the model uses a stratified sampling strategy to divide the dataset into training, validation, and test sets, and minimizes the error through the following objective function:

7 $${\text{Loss}} = \frac{1}{n}\sum\limits_{{i = 1}}^{n} {\left( {y_{i} - \hat{y}_{i} } \right)^{2} } + \lambda \left\| \Theta \right\|^{2}$$

where the regularization term $\lambda \left\| \Theta \right\|^{2}$is used to prevent overfitting.

### Evaluation metrics

To comprehensively evaluate the performance of the ABC-LSTM model in the business performance assessment of SMEs in cross-border e-commerce, the study designs multiple evaluation metrics from various dimensions.

The Mean Absolute Error (MAE) is an indicator that measures the absolute deviation between the predicted values and the actual values. Its formula is:

8 $${\text{MAE}}=\frac{1}{n}\sum\limits_{{i=1}}^{n} {\left| {{y_i} - {{\hat {y}}_i}} \right|}$$

where y_i_ and ${\hat {y}_i}$ are the actual value and predicted value of the i-th sample, respectively, and n is the number of samples. MAE directly reflects the average level of prediction error, and it is highly interpretable and intuitive. The Mean Bias Error (MBE) is used to assess the systematic bias in the predicted values. Its formula is:

9 $${\text{MBE}}=\frac{1}{n}\sum\limits_{{i=1}}^{n} {\left( {{y_i} - {{\hat {y}}_i}} \right)}$$

The Coefficient of Determination (R²) measures the goodness of fit of the model to the data. Its formula is:

10 $${R^2}=1 - \frac{{\sum\limits_{{i=1}}^{n} {{{({y_i} - {{\hat {y}}_i})}^2}} }}{{\sum\limits_{{i=1}}^{n} {{{({y_i} - \bar {y})}^2}} }}$$

where $\bar {y}$ is the mean of the actual values. The value of R^2^ ranges from 0 to 1, with a value closer to 1 indicating a better fit of the model to the data. The Time-Dependent Error (TDE) further examines the model’s prediction performance along the time dimension. Its formula is:

11 $${\text{TDE}}(t)=\frac{1}{k}\sum\limits_{{i=t}}^{{t+k - 1}} {\left| {{y_i} - {{\hat {y}}_i}} \right|}$$

where t represents the time point, and k is the length of the time window. TDE(t) represents the average absolute error within the time window around t. TDE can reveal fluctuations in prediction performance during specific time periods, making it suitable for analyzing the dynamic prediction capability of the model for the performance of cross-border e-commerce enterprises.

## Research results and analysis

### Descriptive statistics

As shown in Table 3, in terms of profitability, the mean values of ROA and ROI are 0.122 (0.045) and 0.177 (0.044), respectively, indicating that the sample enterprises are generally at a moderate level in terms of asset and capital profitability. This reflects that the cross-border e-commerce industry is able to maintain a certain level of profitability during its development; however, due to market competition and external environmental uncertainties, some enterprises exhibit significant volatility in their profit performance. In terms of financial stability, the mean value of the Debt-to-Asset Ratio (DAR) is 0.501 (0.124), indicating that the sample enterprises, on average, maintain a moderate level of debt, but there are significant differences in the capital structure between enterprises. Highly leveraged enterprises may rely excessively on external capital during financing, increasing financial risk, while enterprises with lower leverage may maintain relatively stable financial health through cautious capital utilization. The mean value of the Quick Ratio (QR) is 1.752 (0.437), indicating that most enterprises have strong short-term solvency. However, a few enterprises may face short-term liquidity issues. The mean value of the Total Asset Turnover (TAT) is 1.242 (0.422), showing that, overall, enterprises have relatively balanced asset operation efficiency, although individual enterprises may have varying performance due to differences in supply chain management efficiency or inventory turnover cycles. In terms of business expansion ability, the mean value of the Revenue Growth Rate (RGR) is 0.085 (0.116), with a minimum value of -0.098. Although the average value shows a positive growth trend, some enterprises with negative growth may be affected by intensified industry competition or market fluctuations. This highlights the need to focus on improving market sensitivity and customer satisfaction to reverse the downward business trend.

**Table 3:** Descriptive statistics results.

	Count	Mean	SD	Min	Max
ROA	160	0.122	0.045	0.051	0.198
ROI	160	0.177	0.044	0.101	0.249
DAR	160	0.501	0.124	0.304	0.697
QR	160	1.752	0.437	1.022	2.5
TAT	160	1.242	0.422	0.527	1.985
RGR	160	0.085	0.116	-0.098	0.299

### Model performance evaluation and comparative analysis

#### Performance evaluation

**Fig. 1:**
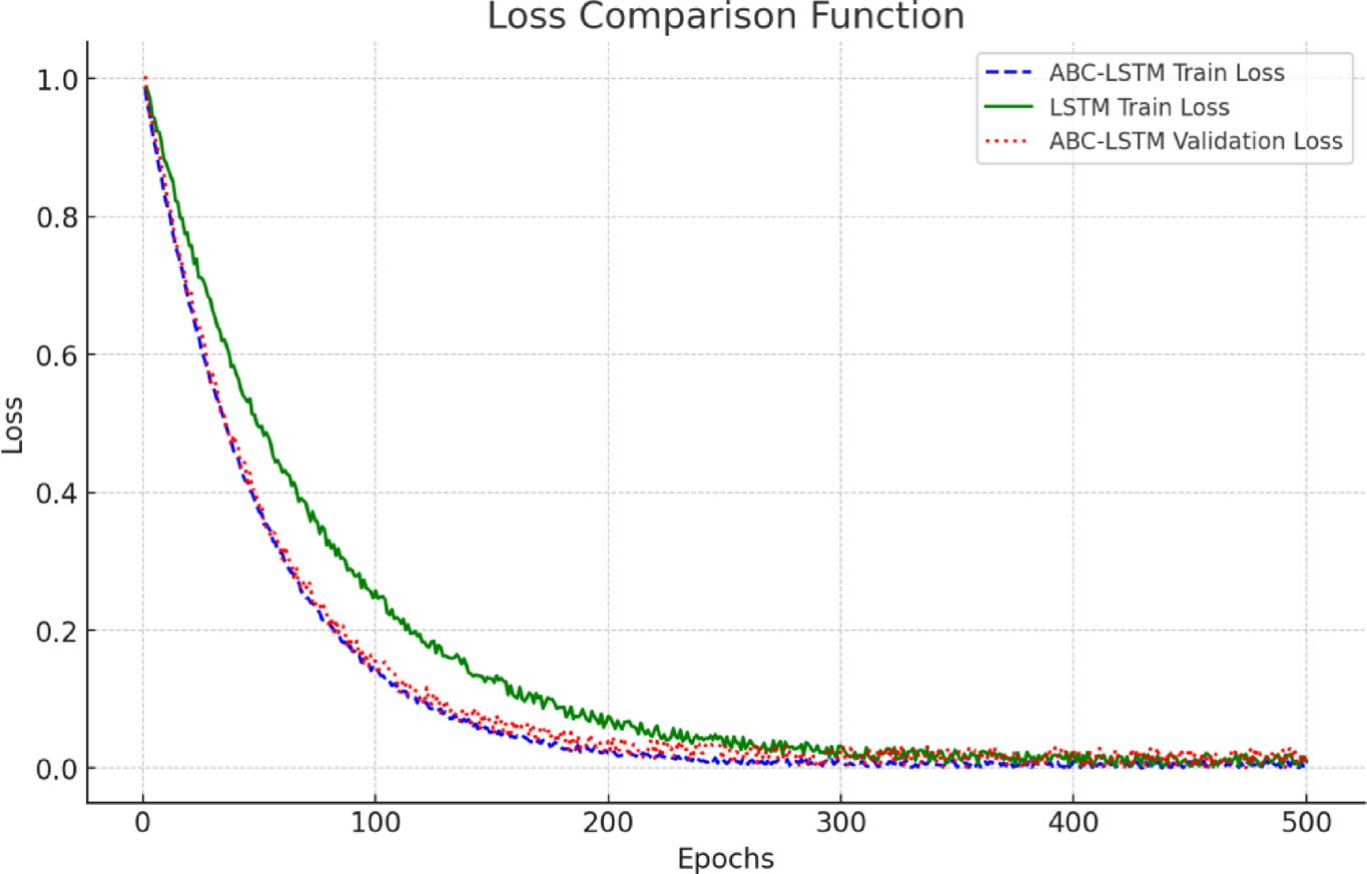
Comparison of model training and validation loss.

Figure 1 shows the training and validation loss curves of the ABC-LSTM model and the traditional LSTM model. In the initial phase, both models have relatively high loss values, which result from the random initialization of the models. As the number of training epochs increases, the loss value of the ABC-LSTM model decreases rapidly and converges significantly faster than the traditional LSTM within the first 100 epochs. This is mainly due to the global and local search capabilities of the Artificial Bee Colony (ABC) algorithm, which effectively optimizes the hyperparameter configuration. At the 50th epoch, the training loss of the ABC-LSTM model had already decreased to 0.399, while the traditional LSTM remained at 0.503, highlighting the significant advantage of the optimization strategy in improving convergence efficiency. In the later training stages, the ABC-LSTM model stabilized around the 300th epoch, with the loss dropping below 0.01 and maintaining low volatility. In contrast, the traditional LSTM still exhibited slight fluctuations even after 400 epochs, indicating a tendency to fall into local optima or overfitting issues when handling complex time-series data. In contrast, the traditional LSTM model exhibits slower loss convergence and shows significant fluctuations in the later stages, indicating that it tends to get stuck in local optima or overfitting issues when dealing with complex time-series data. On the other hand, the validation loss of the ABC-LSTM model is close to the training loss curve, demonstrating its good generalization ability, while the traditional LSTM’s validation loss is notably higher than its training loss, indicating poor prediction performance on unseen data. Overall, the ABC-LSTM model outperforms the traditional LSTM model in both convergence speed and generalization performance, proving the effectiveness of incorporating intelligent optimization algorithms to enhance model performance.

#### Comparative analysis

In this study, GA-LSTM, XGBoost, and LSTM are chosen as comparison models based on their representativeness and applicability. GA-LSTM optimizes the LSTM hyperparameters using a genetic algorithm to improve model performance, but it has limited local search capability. XGBoost, based on gradient boosting trees, performs non-linear regression efficiently, but its performance in modeling long-term dependencies is limited. LSTM, as the baseline model, captures long-term dependency information through gating mechanisms, but its performance depends on hyperparameter tuning. By comparing these three models, the effectiveness of the artificial bee colony algorithm (ABC) in enhancing LSTM’s performance can be comprehensively validated, showcasing the advantages of deep learning models in complex feature modeling tasks.

**Fig. 2:**
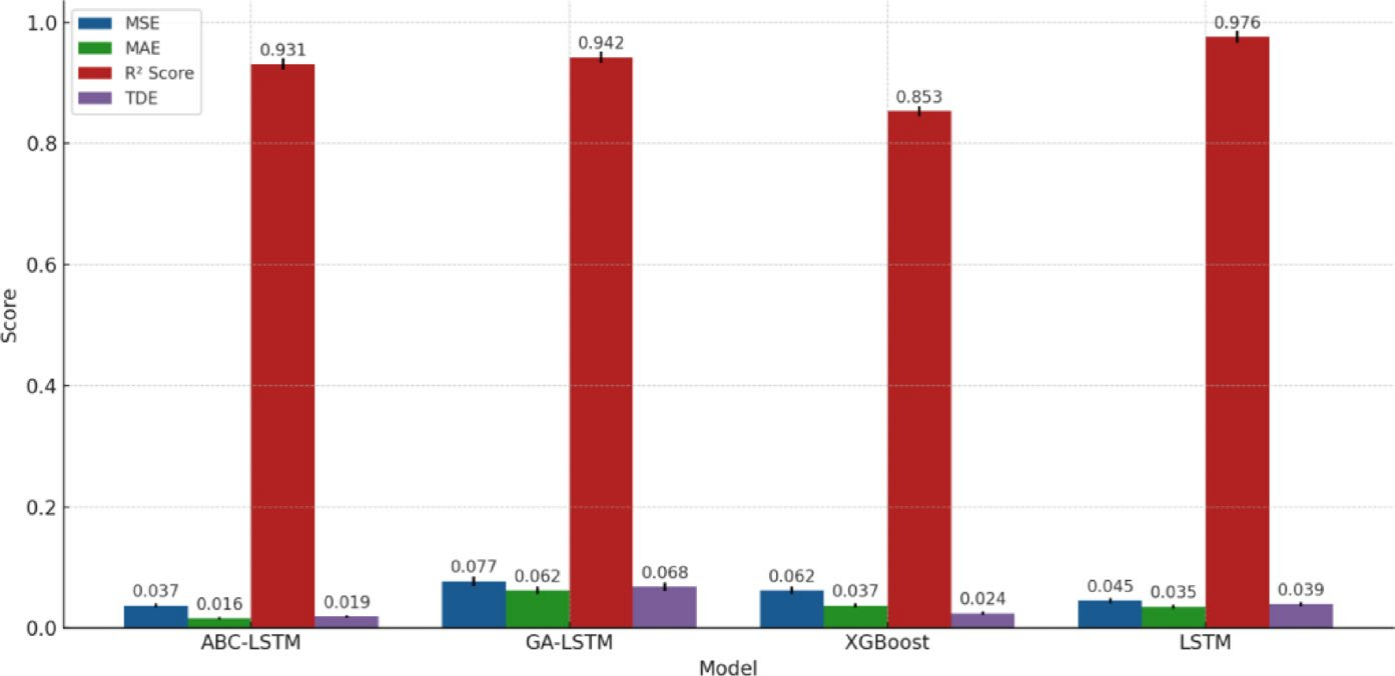
Model performance comparison.

Figure 2 presents the performance metrics of ABC-LSTM, GA-LSTM, XGBoost, and LSTM. In terms of MSE and MAE, ABC-LSTM’s MSE is 0.037, and MAE is 0.016, significantly lower than GA-LSTM (MSE = 0.077, MAE = 0.062) and XGBoost (MSE = 0.062, MAE = 0.037), indicating its superior performance in overall and absolute error control. This result reflects how the artificial bee colony algorithm improves model convergence efficiency through a balance of global search and local exploration during hyperparameter optimization, preventing parameters from getting trapped in local optima. Additionally, ABC-LSTM’s TDE is 0.019, much lower than GA-LSTM’s 0.068, suggesting that its prediction error fluctuations are smaller across different time windows, thus exhibiting higher stability.

In comparison, although GA-LSTM performs well with an R² of 0.942, indicating good overall data fitting, its TDE is as high as 0.068, suggesting local instability in complex time series. While XGBoost improves non-linear regression capability through an ensemble learning framework with tree structures, its R² is only 0.853, significantly lower than deep learning models, validating its limitations in modeling long-term dependencies. LSTM has an R² of 0.976, but its MAE and TDE are 0.035 and 0.039, respectively, indicating a tendency for overfitting during training, which results in slightly poorer generalization performance.

Therefore, ABC-LSTM, through the optimization of the artificial bee colony algorithm, not only achieves higher accuracy in error control but also excels in the stability of time-dependency errors, demonstrating the effectiveness of intelligent optimization algorithms in improving model performance in complex time series prediction tasks. As a result, ABC-LSTM achieves a better balance across multiple performance metrics.

### Performance evaluation

Based on the prediction data analysis in Table 4, the current performance of small and medium-sized cross-border e-commerce enterprises in China shows significant stratification. Specifically, it reflects the coexistence of high-performance enterprises’ strong competitiveness and the challenges faced by low-performance enterprises. Among the 10 enterprises, 30% perform excellently, with their comprehensive predicted values exceeding 3.0. These high-performance enterprises include Company A (3.313), Company E (3.103), and Company J (3.028), typically characterized by excellent resource integration capabilities, efficient supply chain management, and stable market expansion abilities. In terms of profitability and asset operating efficiency, these enterprises show high balance and stability in their predicted data, likely due to their leading positions in the global market and efficient internal management coordination. These three enterprises adopt operation models such as “independent website + overseas warehouse” or “proprietary platform + overseas warehouse,” which grant them greater autonomy over distribution channels and inventory control. This enhances their logistics responsiveness and customer retention. Their main product categories—home goods and baby electronics—are characterized by relatively stable market demand and high repurchase rates, laying a solid foundation for sustained performance growth. Moreover, all three enterprises are located in cross-border e-commerce clusters such as Guangdong and Zhejiang, where regional supply chain support and export infrastructure are well-developed, further strengthening their operational resilience.

60% of the enterprises’ predicted total values fall between 2.0 and 3.0, indicating medium performance. These enterprises demonstrate some competitiveness, but volatility in certain indicators or regional disadvantages limit their further development. These enterprises may not have fully leveraged their potential in asset operation efficiency or business expansion capabilities, and under external competitive pressures, they need to optimize their market strategies and operational management. This type of enterprise typically adopts a platform-based B2C operation model—for example, Companies B and G rely on platforms such as Amazon and AliExpress for sales. While these platforms offer traffic advantages, they also impose significant constraints through platform regulations, resulting in relatively weak bargaining power for the enterprises. Their main product categories, such as apparel accessories and stationery, are susceptible to seasonal trends and consumer preferences, leading to pressures in sales volatility and inventory turnover. Additionally, some of these enterprises are located in regions like Jiangsu and Fujian, where industrial chain support is less developed compared to Guangdong, thereby limiting their overall operational efficiency. As a result, under high external competitive pressure, these enterprises often face a greater need to optimize their market strategies and operational management.

Low-performance enterprises account for 20%, with their predicted total values below 2.0, including Company D (1.946) and Company F (1.637). These enterprises perform poorly across multiple dimensions, with significant weaknesses in business expansion ability and financial stability. Company F ranks last with a total score of 1.637. Its main products—jewelry and footwear—are characterized by low added value and intense competition. The company relies on platforms such as Etsy, facing typical challenges such as a fragmented customer base and high return rates. Company D, on the other hand, focuses on household storage products. Although the market size is considerable, it faces issues of severe product homogeneity and frequent price wars on the AliExpress platform. Moreover, the company lacks independent brand development capabilities, suffers from low resource integration efficiency, and bears a heavy financial burden. Neither company has adopted self-operated channels nor established overseas warehouses, putting them at an inherent disadvantage in order fulfillment and customer responsiveness. These factors may be key contributors to their persistently poor performance. This stratification phenomenon reveals the diversified characteristics of small and medium-sized cross-border e-commerce enterprises in China. High-performance enterprises provide a good development model for the industry, while medium- and low-performance enterprises need to catch up with industry leaders by optimizing management models, improving operational efficiency, and strengthening technological innovation.

**Table 4:** Cross-border e-commerce enterprise performance prediction data.

Enterprise	Profitability	Asset operating efficiency	Business expansion ability	Financial stability	Predicted total value	Rank
Company A	0.19	1.802	0.122	1.199	3.313	1
Company B	0.166	1.503	0.103	1.121	2.893	5
Company C	0.153	0.896	−0.018	1.013	2.044	8
Company D	0.135	1.002	0.013	0.796	1.946	9
Company E	0.167	1.797	0.114	1.192	3.103	2
Company F	0.127	0.704	−0.054	0.86	1.637	10
Company G	0.147	1.297	0.122	1.094	2.66	6
Company H	0.134	1.203	−0.027	1.035	2.345	7
Company I	0.177	1.6	0.11	1.105	2.992	4
Company J	0.168	1.7	0.095	1.065	3.028	3

## Discussion

This study systematically evaluated the business performance of small and medium-sized cross-border e-commerce enterprises from 2020 to 2023, using a hybrid model based on Artificial Bee Colony (ABC) optimization and Long Short-Term Memory (LSTM) neural networks. The results show that the ABC-LSTM model significantly outperforms comparison models across multiple performance metrics, demonstrating its exceptional ability to handle high-dynamic data. Moreover, this method not only effectively assesses the business performance of cross-border e-commerce enterprises but also uncovers patterns in performance stratification, providing scientific evidence for SMEs to develop targeted strategies. This paper not only enriches the theoretical research on business performance evaluation but also provides important practical guidance for scientific management in the cross-border e-commerce sector, highlighting the tremendous potential and value of intelligent algorithms in complex dynamic environments.

This paper draws on existing research on time series prediction models and innovatively combines the Artificial Bee Colony optimization algorithm with LSTM, expanding the application scenarios of time series prediction methods. Zou et al.^35^ found that LSTM effectively captures the long-term dependency characteristics of time series data and performs excellently with non-linear dynamic changes in energy consumption prediction. Consistent with their findings, this study also utilizes LSTM for time series modeling and validates its effectiveness in predicting cross-border e-commerce enterprise performance. However, this paper introduces the artificial bee colony algorithm to optimize the hyperparameters of LSTM, overcoming the traditional LSTM’s dependence on parameter selection and significantly improving the model’s convergence efficiency and prediction stability. Chen & Zhou^36^ proposed that combining Genetic Algorithms (GA) with LSTM excels in financial data prediction and demonstrates strong generalization capability for non-stationary series modeling. This study also adopts the hybrid model approach by combining optimization algorithms with deep learning. However, unlike GA, which primarily optimizes parameters through genetic mechanisms, ABC algorithms achieve a balance between global search and local optimization by simulating bee foraging behavior. This allows for faster and more stable identification of optimal hyperparameter combinations. By introducing this more efficient optimization algorithm into the enterprise performance prediction domain, this study addresses the inefficiency issues associated with traditional optimization methods. Liwei et al.^37^ explored the application of intelligent algorithms in manufacturing industry performance evaluation and proposed a financial forecasting method based on the XGBoost model, finding that intelligent algorithms can effectively handle high-dimensional, non-linear data, significantly enhancing prediction accuracy. However, their research mainly focused on traditional manufacturing industries, where business models are relatively stable, and data volatility is low. While their findings validated the applicability of intelligent algorithms in business performance evaluation, they did not address the adaptability challenges in high-dynamic environments. In contrast, this study applies the ABC-LSTM model to the cross-border e-commerce sector, achieving accurate multidimensional performance prediction for SMEs and reflecting the dynamic business characteristics of these enterprises through a comprehensive indicator system.

The theoretical contributions of this study are reflected on both methodological and application levels. On the one hand, by constructing a hybrid model that integrates the Artificial Bee Colony (ABC) algorithm with Long Short-Term Memory (LSTM), the study effectively enhances the stability and hyperparameter tuning efficiency of deep learning methods in handling complex time-series data, thereby extending the applicability of intelligent optimization algorithms in business performance modeling. On the other hand, by focusing on cross-border e-commerce SMEs—characterized by high uncertainty and data diversity—the study develops a performance evaluation framework that balances accuracy with adaptability, addressing the insufficient attention to highly dynamic industries in existing literature. Moreover, by validating the model’s practical value in decision-making across dimensions such as resource integration, supply chain management, and market expansion, this research strengthens the theoretical linkage between intelligent algorithms and strategic enterprise management, offering a methodological reference for optimizing SME operations in complex environments. However, the study does have some limitations. First, the sample scope is relatively limited, primarily focusing on small and medium-sized cross-border e-commerce enterprises in the eastern coastal regions of China, which may reduce the regional applicability of the conclusions. Second, the selection of evaluation indicators in this study primarily focuses on internal financial and operational dimensions of the enterprises, without incorporating external environmental variables such as macroeconomic fluctuations, changes in cross-border trade policies, or the stability of international logistics channels. This limitation may constrain the model’s ability to respond to external shocks and reduce its predictive adaptability in real-world scenarios.

## Conclusion

Based on the ABC-LSTM model, this study analyzes the performance data of 10 cross-border e-commerce enterprises from 2020 to 2023 and draws the following key conclusions:

First, the study demonstrates that the performance prediction capability of the ABC-LSTM model significantly outperforms traditional models. The model achieves the best performance in terms of Mean Squared Error (MSE) and Mean Absolute Error (MAE), with values of 0.037 and 0.016, respectively. At the same time, the Time Dependency Error (TDE) is significantly lower than that of the comparison models, at only 0.019, indicating that ABC-LSTM offers higher prediction accuracy and stability in dynamic environments.

Second, the business performance of the sample enterprises exhibits significant stratification. High-performance enterprises have a comprehensive forecast value exceeding 3.0, demonstrating outstanding performance. The prediction values of medium-performance enterprises are concentrated between 2.0 and 3.0, with significant fluctuations in certain indicators. Low-performance enterprises have a forecast value below 2.0, with Enterprise F showing significant weaknesses in key dimensions such as business expansion capability (-0.054) and financial stability (0.86).

This study demonstrates the effectiveness of the ABC-LSTM model in evaluating the business performance of cross-border e-commerce SMEs. The model provides quantitative support for enterprises in key areas such as resource integration, supply chain management, and market expansion, thereby enhancing their ability to respond to complex market environments. As a technical tool within enterprise performance management systems, the model can assist SMEs in identifying operational weaknesses, optimizing resource allocation, and formulating scientific strategies, showing strong potential for practical application and promotion. Future research could expand the sample size by including cross-border e-commerce SMEs from central, western, and non-coastal provinces, covering a broader range of development stages, enterprise scales, and industry types. This would allow for a more comprehensive understanding of how regional economic environments and industry structures affect performance, thereby improving the generalizability and external validity of the study’s conclusions. Additionally, future studies may incorporate external macroeconomic and policy-level variables—such as exchange rate fluctuations, international logistics costs, export rebate policies, and overseas market climate indices—into the modeling framework alongside internal business indicators. This would enhance the model’s predictive accuracy and stability in multi-source information environments, strengthening its adaptability to complex dynamic markets and improving its practical explanatory power.

## Data Availability

All data analyzed in this study are available on request from the corresponding author.
